# Hunting for the ultimate liquid cancer biopsy - let the TEP dance begin

**DOI:** 10.1186/s12964-016-0147-9

**Published:** 2016-09-27

**Authors:** Stephan M. Feller, Marc Lewitzky

**Affiliations:** Institute of Molecular Medicine, Martin-Luther-University Halle-Wittenberg, Halle (Saale), Germany

## Abstract

Non-protein coding RNAs in different flavors (miRNAs, piRNAs, snoRNAs, lncRNAs, SHOT-RNAs), exosomes, large oncosomes, exoDNA and now tumor-educated platelets (TEPs) have emerged as crucial signal transmitting, transporting and regulating devices of cells in the last two decades. They are also establishing themselves increasingly in the realm of tumor research. We are currently witnessing a mushrooming of candidate entities for diagnostic and prognostic cancer detection and characterization tests that could have a major impact on how this diverse group of diseases is initially spotted and subsequently treated in the near future. But how do the new kids on the block stand up to the more established circulating tumor cells (CTCs) and circulating tumor DNA (ctDNA)? Without question, much earlier disease detection would be expected to save numerous lives. With all these new players around, will we finally win a major battle in the never-ending war against cancer?

## Background

Highly effective early cancer detection could save a huge number of patients from devastating, marginally effective therapies that are commonly accompanied by morbidity and/or followed by early death. Discovering tumors very early on, ideally before metastasis sets in, has therefore been on the minds of many cancer researchers and health care providers alike. After all, it is metastasis that kills the vast majority of cancer patients [1].

Cancers typically start from a single cell. However, with our present day routine methods this single cell usually will have multiplied into a billion or more cancer cells and often will have also evolved into several distinct subclones before the tumor is finally detected.

It is commonly through patient observations and not specific medical tests that initial cancer signs emerge, for example in the form of a lump or some sort of pain. This then sets into motion a series of histological and/or molecular tests to determine the tumor origin and possibly even the disease subtype.

Apart from the somewhat disputed successes of large scale routine mammographies [2], as well as visual skin inspections and Pap smears, current medical practice has fairly little to offer in terms of non- or minimal-invasive early cancer detection procedures.

Manual prostate inspection and PSA determination are fairly crude tools that seem to have no substantial impact on prostate cancer survival rates [3, 4]. Endoscopic inspections of the aerodigestive tract could probably contribute to a significant boost in survival rates of some cancer types, but are by and large ignored as routine screening options for eligible individuals. They come with a low but non-negligible risk resulting from mechanical damages (bleeding, perforation) and anaesthetization complications (for more details see http://www.bsg.org.uk/pdf_word_docs/complications.pdf).

Simple, robust analyses of body fluids like blood, saliva and urine would therefore be a quantum leap forward in our probably infinite quest to improve cancer survival rates. These ‘liquid biopsies’ [5], would have to detect cancer cells or their various products with great reliability to provide a practical, convenient and possibly only moderately costly expansion of our limited present day repertoire of cancer detection methods.

Until now, such new test forms are mostly under development in various research laboratories and not widely applied in routine medical practice. This might, however, change in the next years. There are many candidates in the form of molecules and macromolecular assemblies to be considered, which could drive these changes.

## Main text

### Cells and extracellular vesicles

The first category of candidates are the by now ‘classical’ circulating tumor cells (CTCs) [6, 7] and circulating DNA fragments (ctDNAs) [8, 9]. They are currently explored in several dozen clinical studies (see e.g. ClinicalTrials.gov for most recent details). Probably the most exciting new development in this area is the very recent finding that ctDNA seems to have a major role to play as a prognostic marker of surgery success of stage II colon cancer resections [10].

**Fig. 1:**
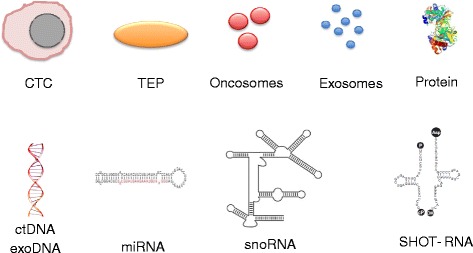
Cells, vesicles and molecules in liquid biopsies that can be queried for information about cancers (selected examples)

In addition, we are witnessing at present the surfacing of a whole zoo of novel molecule and vesicle classes that promise to provide information about cancer origins and subtypes. Some of them will be briefly introduced here (see also Fig. 1 for selected examples).

Extracellular vesicles come in many forms, sizes, shapes [11, 12] and their nomenclature and definition is still somewhat fuzzy, since universal exosomal markers are still missing [13–15]. Several curated databases like ExoCarta [16] and EVpedia [17] are collecting information on their molecular components and characteristics. Exosomes are endocytic secretions that can be generated by many cell types, at least in vitro, and represent, at least for now, the most prominent extracellular vesicle group. They are detectable in many body fluids, form a diverse bunch of membrane vesicles ranging in size from ca. 30 to 150 nm and can be isolated by several different methods, including immunoaffinity capture and ultracentrifugation [18–20]. Composed of proteins, various RNAs and lipids, exosomes carry inside and on their surface a rich load of information that can be deciphered by molecular readout methods like RNA sequencing and proteomic approaches. Exosomes have just been published as suitable leads for the early detection of pancreatic cancer and the cell surface proteoglycan glypican-1 has been proposed as a cancer exosome marker [21]. Moreover, exosomes are believed to contain double-stranded DNA from their generating cells, which has been christened exoDNA [22, 23], but this finding requires further validation and exploration before a possible role of this DNA as biomarker can be considered. Fairly recently, exosomes have been implicated as major players in cancer development, for example, in driving organ-specific metastasis [24, 25]. It is thus to be expected that exosomes will be a major focus of biochemical and functional cancer studies in coming years.

Oncosomes, originally described 2008 in gliomas as vehicles for intercellular transfer of an oncogenic EGFR variant [26], are much larger in size (ca. 1–10 μm) and differ considerably in their composition from exosomes and other extracellular vehicles [15, 27]. They are believed to be generated by membrane blebbing in late stage cancer disease. In prostate cancer cells, oncosome production was reportedly stimulated by EGFR activation and elicited in recipient cells an increase in tyrosine phosphorylation and Akt signalling, thereby altering the tumor microenvironment and contributing to disease progression [28]. A new study has further defined the generation, density, composition and cargo of oncosomes [15]. It documents the enrichment of cytokeratine 18 (CK18) as a molecular marker of oncosomes and links enhanced oncosome shedding to silencing of the cytoskeletal formin protein DIAPH3.

### A flourishing superfamily of non-protein coding RNAs

The realization that our genome is not mostly composed of ‘junk DNA’ and that this term predominantly reflected our own ignorance, has led to a profound shift in research activities of scientists focusing on how genomes and their products are regulated. Non-coding RNAs have consequently gained a very solid foothold in most of cell biology research and particularly in human disease studies [29–31].

MicroRNAs (miRNAs), small ribonucleotide oligomers typically composed of ca. 22 nucleotides, are the most intensely studied group of these non-coding RNAs. They serve as repressors of mRNA translation into proteins [32] in a plethora of biological contexts. Many recent reviews have described their generation and functions in considerable detail (for two recent examples see [33, 34]). miRNA involvement in the manifestation and progression of most diseases, ranging from neurological disorders to autoimmunity, inflammation, infections, cardiovascular problems, metabolic pathologies and malignancies is also very well established [35–40]. Over 1900 miRNAs with critical functions have been currently described [41]. miRNAs can act as oncogenes or as tumor suppressors, depending on the specific tissue and cell type context and miRNA polymorphisms have been associated with cancer development risks. Their utility as clinical diagnostics has, however, so far been severely limited by poor data reproducibility. The generation of data in the wet lab and the bioinformatics data processing urgently require optimization and better standardization [41].

Since miRNAs have become a major area of attraction for researchers at the beginning of this millennium [42] over 40,000 publications have investigated the many facets of these fascinating regulatory systems and miRNAs are still getting the lion share of attention until now; but a flurry of additional families of non-protein coding RNAs are emerging in the more recent literature.

Small nucleolar RNAs (snoRNAs) have long been viewed as ‘boring’ housekeeping components of the cell. Hundreds of them have been detected so far. As their name indicates, they live in the nucleolus, the ribosomal RNA producing factory of the cell, form complexes with proteins and aid in the processing of rRNAs [43]. Interestingly, in the last years various groups have reported additional functions of snoRNAs, which link them to the control of cell fates and tumorogenesis [44]. Some snoRNAs have been detected in plasma and could have potential as diagnostic as well as prognostic circulating biomarkers, for example in non-small cell lung cancer (NSCLC) [45, 46]. Larger studies that confirm and extend the initial reports are still needed to validate certain promising snoRNA candidates.

Long non-coding RNAs (lncRNAs), are a highly diverse group of thousands of RNAs implicated in cancer invasion and metastasis, as well as many other diseases [47–49]. For an RNA to be entered into this group, a somewhat arbitrary size threshold of more than 200 nucleotide in lengths has been set. At least a few lncRNAs have been detected in human plasma so far and may have relevance for diagnostic or prognostic assays [50, 51], but clearly much more work is needed to gain a robust understanding of the potential and limits of lncRNAs for cancer detection, classification and treatment.

PIWI protein-interacting RNAs (piRNAs) are single-stranded molecules composed of ca. 26–31 nucleotides and found throughout the animal kingdom [52]. A major, if not the major, function of piRNAs is the immobilization of transposons, particularly in the context of spermatogenesis [53]. Whether piRNAs are really drivers of cancer development or ‘passengers’ still remains to be determined [54]. Although they can be detected as a small fraction of the various RNAs isolatable from plasma-derived exosomes [55], it is far from clear whether they will have any diagnostic value in clinical settings.

Only last year, a new group of RNAs has shot onto the scene and gained significant attention immediately: sex hormone-dependent tRNA-derived RNAs (SHOT-RNAs). Generated from mature tRNAs, they are constitutively present in sex hormone-dependent breast and prostate cancer cells [56]. The ribonuclease angiogenin has been implicated in their biogenesis and sex hormones and their receptors were shown to enhance the generation of SHOT-RNAs. Their potential as diagnostic and/or therapeutic targets, if any, remains to be explored.

### Developing analyses of circulating macromolecules, vesicles and cells towards clinical routine applications

CTCs from solid cancers have proven fairly difficult to work with, for all but a few specialist laboratories. CTCs are usually exceedingly rare and their vitality at the time of isolation is often uncertain. Despite nearly two decades of increasing scrutiny ([57, 58], methods to actually culture them ex vivo after isolation are just being developed [59]). Nevertheless, successful RNA sequencing from single CTCs of prostate cancer patients has been reported [60], implicating non-canonical Wnt signaling in mediating anti-androgen resistance. This indicates that it may be possible to omit the ex vivo amplification step for CTCs, at least for some types of analyses. CTC plasticity is, however, another significant worry and clearly needs further elucidation. Along these lines, EpCAM-positivity cannot be considered a robust feature of CTCs anymore [61].

Whole exome and whole genome sequencing experiments of DNA from tissue biopsies was and is certainly enormously helpful to gain much deeper knowledge about the molecular mechanisms and heterogeneity of virtually all cancer types. These are by now also possible using single CTCs [62–64]. CTCs could thus, at least in principle, provide some useful information on the (sometimes changing) polyclonality of tumors. Multiple standard biopsies that are otherwise required to analyze the clonality of tumors, are difficult or impossible to obtain for certain tumor types, so alternative options would be most welcome. Until now, however, it is not really clear how well tumor polyclonality is really reflected in CTCs and whether the current methods of CTC analysis are developed far enough to make this a practical option.

Alternatively, cancer-relevant miRNAs in liquid biopsies, the so called ‘Oncomirs’, [40, 65] might be analyzed. But even there, the required technology is just being developed and still far from being ready for daily routine usage [66, 67].

In general, it can probably be expected that costly ‘-omics’ approaches will not be the main road forward for new diagnostic cancer tests, at least when it comes to broad clinical implementation, even for large comprehensive cancer centers (CCCs). While ‘-omics’ technologies clearly very important for basic and translational research, it is highly desirable to generate more focused analyses, no matter what the material (s) for the analyses will eventually be. Limited sets of molecular markers, analyzed by simple, low cost array type (micro-) chips and automatically read and evaluated by standardized, automatic machines would seem to be the method of choice to lead the way into daily routine application.

### Tumor educated platelets (TEPs)

It might be at first counterintuitive to assume that highly chaotic entities like tumor cells could ever be ‘educators’ of anything. Obviously this term is used quite loosely here and a slightly better one for the recently observed phenomenon of tumor-influenced platelets [68–70] might be ‘tumor-conditioned platelets’.

It is, however, also undoubtedly clear that cancer cells impact very significantly on several other cell types, ranging from fibroblasts (carcinoma-associated fibroblasts (CAFs), tumor-associated fibroblasts (TAFs)) and various immune cells, like tumor- associated macrophages (TAMs) and tumor-infiltrating lymphocytes (TILs) to endothelial cells. Molecular studies of the relationships between different cell types within a tumor mass or a metastatic lesion are currently under way and provide evidence for complex, formerly unknown relationships at a rapid pace [71–73].

By comparison, analyzing effects of tumor cells on platelets has been somewhat neglected until now. After all, platelets are merely sheddings of megakaryocytes in the bone marrow that do not contain a nucleus and possess a reduced repertoire of proteins, RNAs etc. Their main normal function is hemostasis, i.e. plugging vascular holes after physical injuries.

However, cancer progression can be accompanied by platelet activation and venous thromboembolisms (VTEs). In addition, platelets can secrete growth factors that can influence cell growth and angiogenesis. They are also able to attract other blood cells like monocytes and granulocytes, which may help to create a pro-metastatic microenvironment [74–76].

The latest TEP study by Best and colleagues [70] is highly intriguing. Based on extensive platelet RNA analyses of more than 280 individuals (ca. 22 million reads per sample) they were able to distinguish healthy persons from cancer patients with more than 95 % accuracy and could even provide some information on likely tumor locations for some major tumor types. Five-thousand non-coding and protein-coding RNAs were detectable in pictogram quantities of RNA, corresponding to platelet numbers that can be found in a single drop of blood. The global RNA deregulation observed was massive, with over 2000 changes observed.

Interestingly, prominent tumor-driving mutations (EGFR, Her-2, c-Met, H-Ras, PI3KCA) were also accurately reflected in the results obtained from platelet RNA sequencing and bioinformatics processing of the resulting data. This finding could have major implications for future clinical trials and therapy regimens [77].

While the tumor type was the predominant factor for the actual platelet conditioning, tumor metastasis did not significantly impact on them when compared to samples from patients without metastasis, which is somewhat surprising.

It should be pointed out that the majority of cancer patients had metastatic disease (189 of 228), which usually corresponds to a high tumor mass burden, i.e. many billions of tumor cells, although patients with localized disease (albeit unspecified tumor volume) were also correctly classified. Furthermore, the average age of the healthy donors was significantly lower than that of the cancer patients.

- So are TEPs going to run CTCs out of business?
  They are obviously easy to purify, infinitely more abundant and much less likely to die while moving through the vascular system. So this may sound like a done deal, yet it is probably not possible to give a clear-cut answer at present. TEPs will need to be scrutinized much further until a number of questions have been answered.
  Some of the most pressing ones are:
- Will other researchers be able to recapitulate the initially reported results time and time again? In other words, how robust is this method?
- Will it become possible to simplify the analyses to a degree that becomes feasible in daily routines, for example by focusing on specific subsets of RNAs without loosing accuracy?
- How much will platelet RNAs from non-cancerous individuals vary depending on their age or the presence of various non-malignant diseases?
- What is the sensitivity threshold for the cancer patient studies? Clearly, a single tumor cell should not be able to ‘educate’ a vast number of platelets. So how many cancer cells are needed before conditioning effects become apparent? Many billions?
- How does polyclonality factor into this?
- Some well-known cancer mutations, mentioned already above, have been detectable through TEP RNA analyses. But how many other, rarer mutants and variants will be detectable robustly?
- And what about the highly complex epigenetic patterns? How will they be reflected in TEPs?

The exciting initial work described by Best and colleagues is bound to be followed up soon by studies of larger cohorts of individuals with specific tumor types, patients with tumors of different sizes etc.

## Conclusions

In summary, a new battle has just begun, and there is certainly some hope for success. We now know our old enemy cancer much better than only a decade ago, though it is bound to still have a few more tricks up its sleeves. Whether it will actually become possible to snatch a few more mortals away from him for some time by utilizing liquid biopsies, in the form of TEPs and other marker vesicles or nucleic acids, remains to be determined.

## Abbreviations

CTC: Circulating tumor cells
ctDNA: Circulating tumor DNA
exoDNA: Exosomally-derived DNA
lncRNA: Long non-coding RNA
miRNA: Micro RNA
piRNA: PIWI protein-interacting RNA
SHOT-RNA: Sex hormone-dependent tRNA
snoRNA: Small nucleolar RNAs
TAF: Tumor-associated fibroblast
TAM: Tumor-associated macrophage
TEP: Tumor educated platelet
TIL: Tumor infiltrating leukocyte
VTE: Venous thromboembolism
